# Carbon Derived from Jatropha Seed Hull as a Potential Green Adsorbent for Cadmium (II) Removal from Wastewater

**DOI:** 10.3390/ma6104462

**Published:** 2013-10-09

**Authors:** Masita Mohammad, Zahira Yaakob, Siti Rozaimah Sheikh Abdullah

**Affiliations:** Department of Chemical and Process Engineering, Faculty of Engineering and Built Environment, Universiti Kebangsaan Malaysia (UKM), Bangi 43600, Selangor, Malaysia; E-Mails: zahirayaakob65@gmail.com (Z.Y.); rozaimah@eng.ukm.my (S.R.S.A.)

**Keywords:** adsorption activity, carbon, jatropha seed hull, cadmium, kinetic and isotherm model

## Abstract

Carbon from jatropha seed hull (JC) was prepared to study the adsorption of cadmium ions (Cd^2+^) from aqueous solutions under various experimental conditions. Batch equilibrium methods have been used to study the influences of the initial metal ion concentration (0.5–50 ppm), dosage (0.2–1 g), contact time (0–300 min), pH (2–7), and temperature (26–60 °C) on adsorption behavior. It has been found that the amount of cadmium adsorbed increases with the initial metal ion concentration, temperature, pH, contact time, and amount of adsorbent. A kinetic study proved that the mechanism of Cd^2+^ adsorption on JC followed a three steps process, confirmed by an intraparticle diffusion model: rapid adsorption of metal ions, a transition phase, and nearly flat plateau section. The experimental results also showed that the Cd^2+^ adsorption process followed pseudo-second-order kinetics. The Langmuir and Freundlich adsorption isotherm models were used to describe the experimental data, with the former exhibiting a better correlation coefficient than the latter (*R*^2^ = 0.999). The monolayer adsorption capacity of JC has been compared with the capacities of the other reported agriculturally-based adsorbents. It has been clearly demonstrated that this agricultural waste generated by the biofuel industry can be considered a potential low-cost adsorbent for the removal of Cd^2+^ from industrial effluents.

## 1. Introduction

*Jatropha curcas* L. is a multipurpose shrub or small tree that mainly grows in the wild and has many useful attributes and numerous uses. It is economically important in the production of oil. The crops belong to the family Euphorbiaceae, which is comprised of approximately 8000 species belonging to 321 genera [1]. Recently, it has also been planted as a commercial crop due to gradually increasing interest in biodiesel [2]. In Malaysia, wild *Jatropha curcas* L. is known as *Jarak pagar* and is found in Peninsular Malaysia and East Malaysia. The plant has low moisture demands, low fertility requirements, and tolerance to high temperatures, which make it suitable to grow in tropical climates [3]. The jatropha seed hull is the wall of the seed, known as a seed coat or testa, the true seed lies within the kernel with a very thin layer of endosperm. The jatropha seed hull has a hard and blackish hull similar to the sunflower seed hull but is different in size and shape and lacks white stripes [4].

Jatropha seed hull, which is an agricultural waste generated by the biofuel industry, can be considered as a potential low-cost adsorbent for the removal of cadmium (Cd^2+^) ions from industrial effluents. Research has been carried out using untreated seed hull for the adsorption of a dye (malachite green) and heavy-metal ions, such as zinc and cadmium, and it has shown a remarkable adsorption capacity. The adsorption capacity of the jatropha seed hull can reach up to 11.89 mg/g for cadmium, while that of the castor seed hull can reach up to 6.983 mg/g for cadmium. Due to its high lignin content, jatropha hull has the potential to be a good precursor for the production of activated carbon.

Cadmium is a hazardous waste derived from industrial wastewater, which is harmful to the environment and human beings. Cadmium (Cd^2+^) is released into wastewater generated by industries, such as those dedicated to electroplating, cadmium-nickel batteries, phosphate fertilizers, pesticides, mining, pigment, and alloys, and is also released from sewage sludge [5,6,7,8,9,10]. Various treatment processes, such as chemical oxidation, reduction, precipitation, solidification, electrolytic recovery, solvent extraction, membrane separation, ion exchange, and adsorption on activated carbon, are used for the removal of metal ions from wastewater [8,11]. However, specific applications of such methods are sometimes restricted because of technical or economical constraints.

Adsorption is a process in which dissolved species in aqueous solution or gases are attracted to the internal and/or external surfaces of minerals or porous solids, which are known as adsorbent [12,13]. The interactions between gases, liquids, or solids on the surface of a solid or liquid during the adsorption process depends on the nature of the adsorbent and adsorbate. There are two types of adsorption process, namely, physisorption, and chemisorption. Adsorbed molecules are seized by the adsorbent through weak van der Waals forces during physisorption. In chemisorptions, a single layer of molecules, atoms, or ions is attached to the adsorbent surface by chemical bonds. Adsorption is an important phenomenon in many fields within surface science and engineering, such as corrosion, heterogeneous catalysis, chromatography, *etc.*, [14]. This adsorption technique has been widely used for the removal of effluents from wastewater. The techniques are economically feasible for bulk separation processes and can be used for the removal of different types of organic and inorganic pollutants [15,16,17,18].

To date, no study has been carried out on the removal of cadmium metal ions with carbon derived from jatropha seed hull. Many industries in Malaysia, such as the electroplating, plastic, and textile industries, produce wastewater that contains hazardous materials, such as cadmium, zinc, chromium, nickel, and synthetic dyes. The accumulation of these toxic materials in rivers, lakes, and seas is dangerous to humans and many other organisms, and has become a problem for the environment. Currently, activated carbon is commonly used as an adsorbent agent in various industries for the treatment of waste water. In this study, carbon derived from jatropha seed hull without activation used as an adsorbent to minimize its exposure to chemicals substances and reduce activation costs. Moreover, chemically inactive carbon is pursued because its chemically activated counterpart has considerable inorganic content, which may cause environmental contamination and increased the demand for cleaner production [19]. Many researchers have carried out studies using activated carbon derived from many parts of the jatropha plants, such as the cake, husk, and seeds [20,21,22,23]. The characteristics and availability of jatropha seed hull, an agricultural waste, underlie the hull’s potential as a good adsorbent. The study of the efficiency of this aforementioned form of carbon with respect to its adsorption capacity, its characteristics as an adsorbent and its mechanism of adsorption is important to its synthesis.

In this study, we attempt to investigate the effectiveness of jatropha seed hull carbon for the removal of cadmium metal ions through kinetics and equilibrium study. In order to avoid disposal of metal contaminate solid waste and to understand the mechanism of Cd^2+^ adsorption, desorption, and re-usage studies of used jatropha seed hull (JC) has also been done.

## 2. Experimental Section

### 2.1. Process of Producing the Adsorbent

Jatropha seed hull (*Jatropha curcas* L.) obtained from the Biofuel Institute (Universiti Kebangsaan Malaysia), Kuala Pilah, Negeri Sembilan, Malaysia has been used as an adsorbent in this study. The hull was soaked in water and thoroughly washed with water to remove dirt and soluble components. The washed hull was then oven dried at 85 ± 10 °C until reaching a constant weight and crushed into smaller particles. The crushed hull (225 g) was carbonized in a programmable tube furnace (MTI Corp OTF-1200X, Richmond, CA, USA). During the carbonization process, N_2_ gas was introduced to the system at a flow rate of 1000 mL/min. The furnace was programmed in such a way that the heating rate was 10 °C/min and, after reaching the final temperature of 800 °C, was then held at that temperature for 1 h. After cooling, the carbon was rinsed with distilled water several times until the pH was constant. Finally, the obtained product was dried at 100 °C for 24 h, sieved to a particle size of 250–500 μm [60–35 mesh ASTM (American Society for Testing and Materials)], and kept in an air-tight container.

### 2.2. Adsorption Experiment

An adsorption isotherm and kinetics experiment was performed to measure the adsorption of Cd^2+^ on JC from aqueous solution using a batch adsorption equilibrium study. The studies were carried out by shaking 0.4 g of the adsorbent with 100 mL of the solution containing the desired concentrations of the metal ion solution. Cadmium nitrate was used as the salt of the Cd^2+^ adsorbate. The salt used was of an analytical grade (E. Merck, Darmstadt, Germany). A stock solution of 1000 Cd^2+^ ppm was prepared by dissolving 2.74 g of cadmium nitrate tetrahydrate [Cd(NO_3_)_2_ 4H_2_O] in de-ionized water and then diluted to the desired concentration levels ranging from 2 to 50 ppm.

For the equilibrium isotherm studies, 100 mL batches of metal ion solutions with different initial concentrations (2, 5, 10, 20, 30 and 50 ppm) were agitated with 0.4 g of adsorbent using a laboratory shaker (Labwitt ZHWY-304, Shanghai, China) at 160 rpm and 25 ± 2 °C in a series of 200 mL polyethylene bottles for a maximum of 24 h at pH 6. Equilibrium was attained after approximately 7 h.

For the kinetics studies, samples were collected at different time intervals (2, 5, 10, 15, 20, 30, 40, 50, 60, 120, 180, 240, 300, 360, 420, 480 and 540 min) and filtered each time through a 0.45 μm Whatman filter membrane with a syringe. The experiments were carried out by varying the initial metal ion concentrations (2, 5, 10, 20, 30 and 50 ppm), pH (2.5, 3.37, 4.2, 5.12 and 6.2), adsorbent dosage (0.2, 0.4, 0.8 and 1 g), and temperature (26, 35, 45 and 60 °C). For each experiment, the following constant parameters had been used: a dosage of 0.4 g/100 mL, agitation speed of 160 rpm, concentration of 30 ppm, adsorbent size of 0.5 mm, pH of 6, and temperature of 25 °C.

The filtrate solution remaining after the adsorption process and the initial solution before the adsorption process were analysed using a flame atomic absorption spectrophotometer with an air-acetylene flame (Perkin Elmer AAnalyst 800, Shelton, CT, USA). A cadmium hollow cathode lamp was used. The spectral slit width and the working wavelength were 0.7 and 228.8 nm, respectively. The quantity of metal ions adsorbed onto the adsorbent was calculated as the difference between the initial concentration and the concentration at any time, *t*. Each experiment was replicated under identical conditions. The reproducibility of the measurements was within an error rate of 10%.

### 2.3. Desorption Experiment

Hydrochloric acid, HCl, has been chosen as desorption agent in this desorption study. Approximately 0.4 g of Cd^2+^ loaded JC from the previous adsorption experiment was gently washed to remove any unadsorbed Cd^2+^. Then, it was stirred with 100 mL of 0.1 M HCl solution for 24 h. The filtrate was analyzed for desorbed Cd^2+^ ions using Atomic Absorption Spectrophotometer. To test the reusability of the JC, the same adsorbents were washed with distilled water and desorption process were repeated for several times. The concentration of released Cd^2+^ ions form washing process was again analyzed. The amount of Cd^2+^ ions released from the JC is the desorption value of JC. The desorption ratio (%) of Cd^2+^ from JC was calculated by percentage of the deduction amount of Cd^2+^ ions adsorbed on JC to the concentration of Cd^2+^ ions in the desorption medium.

### 2.4. Characterization

The JC was characterized by fourier transform infra-red (FTIR) spectroscopy, (Thermo Scientific Nicolet 6700, Madison, WI, USA) to analyze the organic functional groups present in the adsorbent. The surface morphology and elemental composition of JC, before and after the adsorption of cadmium, were examined with a field emission scanning electron microscope, FESEM-EDX (CARL ZEISS EVO MA10, Cambridge, UK) with gold-coated samples. Elemental composition in terms of carbon, hydrogen, nitrogen, sulphur and oxygen was measured using a CHNSO Analyzer (Thermo Fin 1112 CHNSO-Analyser, Rodano, Italy). The surface composition of the sorbent was determined by energy dispersive X-ray fluorescence (PANalytical Mini Pal 2, Almelo, The Netherlands) analysis. The specific surface area and pore size of JC were measured according to the BET (Brunauer, Emmett, and Teller) method by N_2_ adsorption at 77 K using a BET Micromeritics ASAP 2020 (Norcross, GA, USA). The BET and BJH (Barrett-Joyner-Halenda) methods were used to calculate the surface area and the pore size distribution of JC. The total pore volume was calculated at a relative pressure (*P*/*P*_0_) of 0.99. The particle size range was measured using a Malvern particle size analyzer (Model Master Seizer 2000, Worcestershire, UK).

### 2.5. Data Analysis

The intraparticle diffusion models [24] were analyzed using the Equation (1).

(1) $$q_{t}=K_{id}t^{0.5}+C$$

where *q_t_* is the amount adsorbed at time *t*, *K_id_* (mg·g^−1^·min^−0.5^) is the rate constant of intra-particle diffusion and *C* is the intercept (mg/L).

The percentage of metal ions removed from solution was calculated using the Equations (2) and (3) was used to calculate the metal ion concentration retained in the adsorbent phase.

Percentage removal,

(2) $$\%R=\frac{\left(C_{i}-C_{t}\right)}{C_{i}}\times 100$$

Adsorption capacity, *q_t_* (mg/g),

(3) $$q_{t}=\frac{(C_{i}-C_{t})V}{m}$$

where *C_i_* (mg/L) and *C_t_* (mg/L) are the concentration in the solution at time *t* = 0 and at time *t*, respectively, *V* is the volume of the solution (L) and *m* is the amount of adsorbent (g) added.

The kinetics experimental data were analyzed using pseudo-first-order [Equation (4)] and pseudo-second-order [Equations (5)–(7)].

Lagergren pseudo-first order equation [25],

(4) $$\text{log}(q_{e}-q_{t})=\text{log}q_{e}-\frac{K_{1}}{2.303}t$$

Pseudo-second-order equations [25,26],

(5) $$\frac{\text{d}q}{\text{d}t}=K_{2}{\left(q_{e}-q_{t}\right)}^{2}$$

(6) $$\frac{t}{q_{t}}=\frac{1}{K_{2}q_{e}^{2}}+\frac{1}{q_{e}}t$$

(7) $$h=K_{2}q_{e}^{2}$$

where *q_e_* represent the amount of metal ions adsorbed (mg/g) at the equilibrium time, respectively, *K*_1_ represents the adsorption first-order rate constant (L·min^−1^) and *K*_2_ is the pseudo-second-order rate constant (g·mg^−1^·min^−1^).

The equilibrium isotherm data were analyzed using the Freundlich [Equation (8)] and Langmuir [Equation (9)] isotherm equations.

Langmuir isotherm [27],

(8) $$\frac{1}{q_{e}}=(\frac{1}{K_{L}q_{m}})\frac{1}{C}+\frac{1}{q_{m}}$$

Freundlich isotherm [28,29],

(9) $$\text{ln}q_{e}=\text{ln}K_{f}+\frac{1}{n}(\text{ln}C_{e})$$

where *C_e_* is the equilibrium concentration of metal ions in solution, *K_f_* is the adsorption capacity and *n* is the intensity of the adsorption.

The dimensionless constant separation factor *R_L_* was analyzed using Equation (10).

(10) $$R_{L}=\frac{1}{1+K_{L}C_{0}}$$

where *k_L_* is the Langmuir constant and *C*_0_ is the initial concentration Cd^2+^.

## 3. Results and Discussion

### 3.1. Characterization of Jatropha Seed Hull Carbon

X-ray fluorescence (XRF) analysis showed that JC is mostly composed of calcium (90.2%); the remaining composition consists of trace elements such as iron (1.4%), nickel (0.1%), copper (0.33%) and zinc (0.26%). It revealed the basic content at the surface of the adsorbent, which supports the adsorption process. As an example, high content of calcium is assumed to enhance the adsorption capacity by their reaction with the adsorbate through translocation of the metal ions between the adsorbent surface and the adsorbate. Moreover, it confirms the absence of any particulate of Cd^2+^ attached to the adsorbent particles prior to the adsorption process.

The elemental distribution of JC in terms of CHNO is analyzed by using CHNSO analyzer. Oxygen accounts for 50.11% of the weight of JC, followed by carbon, hydrogen, and nitrogen, which comprise 48.01%, 1.44% and 0.44% of the total composition by weight, respectively. These values prove the present of carbonyl, hydroxyl, amine, and sulphonyl group, which have an affinity with metal ions.

The particle size distribution of JC was in a range of 0.15–1.18 mm. The surface area (Multipoint BET-N_2_) of JC was found to be 186.12 m^2^/g and the internal surface area (t-method) (119.85 m^2^/g) was greater than the external surface area (66.27 m^2^/g). The surface area could be enhanced by some activation methods, such as with acid, alkaline, or thermal activation, which could increase the adsorption rate. The average pore diameter was observed to 30.58 Ǻ and total pore volume was 0.14 cm^3^/g. The adsorbed volume increased with an increase in P/Po, indicating a wider pore size distribution in the JC adsorbent (data not shown). This porous adsorbent exhibited a type II isotherm, characterized by strong fluid-solid interactions [30].

**Figure 1:**
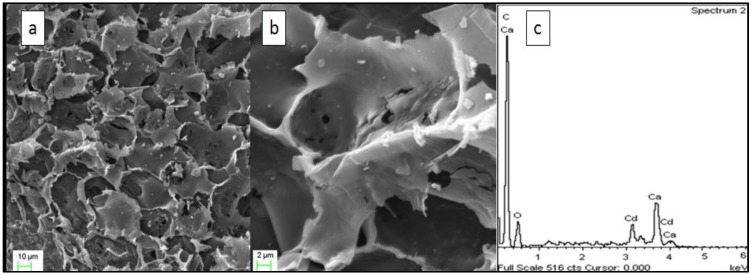
(**a**) FESEM-EDX (Field Emission Scanning Electron Microscopy with Energy Dispersive X-Ray Spectroscopy) images of JC (300 μm) at magnifications of 1000×, (**b**) 5000× and (**c**) EDX image after adsorption.

The SEM images shown in Figure 1 reveal that the external surface is full of cavities, which suggest that JC possesses a high surface area. This morphology is similar to that of acid modified olive stone [31], and char derived from sewage sludge [32]. The results of the EDX (Energy Dispersive X-Ray Spectroscopy) analysis (c) are also shown; the graph shows that the Cd2+ ions were attached to the surface, which possessed a high concentration of calcium, carbon, and oxygen, as mentioned before.

**Figure 2:**
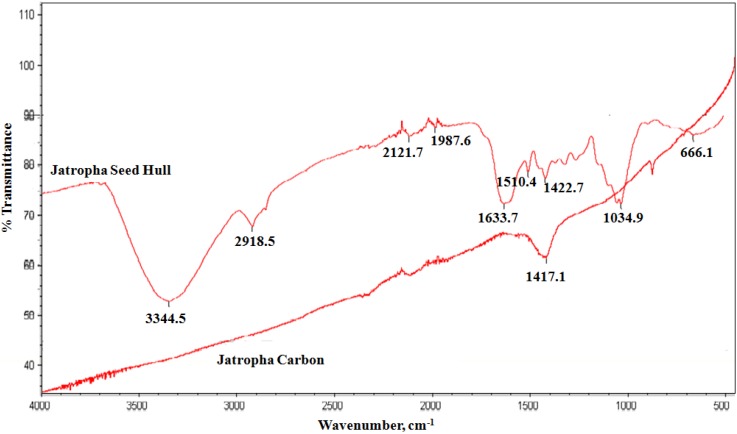
Fourier transform infrared spectroscopy (FT-IR) Spectrum of JC and jatropha seed hull.

Figure 2 shows the flat infrared spectra of JC compared to the infrared spectra of jatropha seed hull. During the carbonization process, most of the functional groups, especially the oxygenated groups, evaporated or were removed; this left only a 1417 cm^−1^ peak, which indicates the presence of C–H and S=O groups. This strong peak might also be due to the high contents of polyphenolic tannins, flavonoids, suberin, *etc.*, in woody plants [33]. Moreover, S=O functional groups generally exhibit very high coordination with heavy metals, especially cations, which could enhance the adsorption capacity of an adsorbent [4]. Aside from these contributions, the low oxygen group density of JC exhibits significant hydrophobic characteristics, which supports the removal of non-ionic solutes from aqueous solution [33].

### 3.2. Kinetic Study

**Figure 3:**
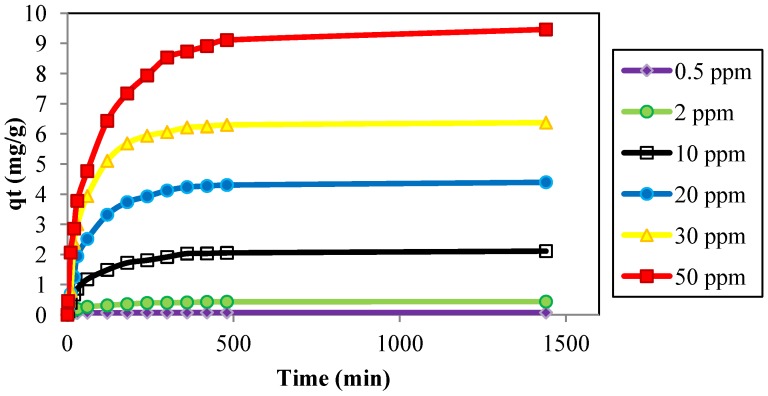
Effect of contact time on the adsorption of Cd^2+^ onto JC at different initial concentrations. (adsorbent size: 0.5 mm, dosage: 0.4g/100 mL, pH: 6, temperature: 25 °C, and agitation speed: 160 rpm).

**Figure 4:**
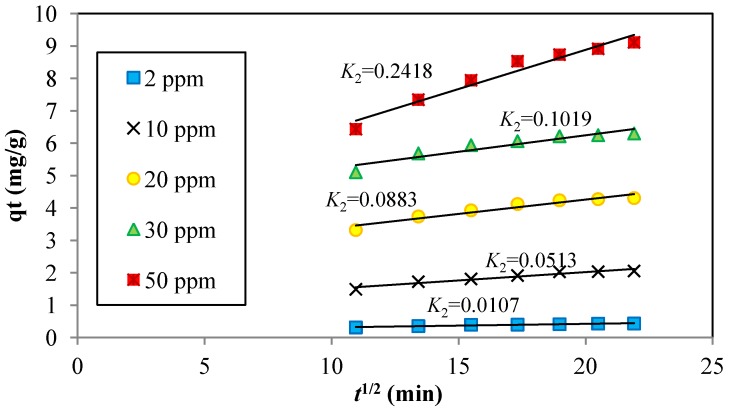
Intraparticle diffusion plots for Cd^2+^ adsorption onto JC at different initial metal ion concentrations. (particle size: 0.5 mm, dosage: 0.4 g/100 mL, pH: 6, temperature: 25 °C, and agitation speed: 160 rpm).

A kinetic study regarding the removal of Cd^2+^ ions using the JC adsorbent was performed by varying the initial metal ion concentrations, dosage, contact time, pH, and temperature. Figure 3 shows that Cd^2+^ adsorption increased with an increase in the initial Cd^2+^ concentration. As the Cd^2+^ concentration in the test solution was increased from 0.5 to 50 ppm, the adsorption capacity of JC for Cd^2+^ increased from 0.075 to 9.46 mg/g. By increasing the metal ion concentration, the adsorption capacity for the adsorbent also increased. This may have been due to the increased rate of mass transfer sequence to the increases of concentration as a driving force [34]. Figure 3 proves that the mechanism of cadmium metal ion adsorption onto JC followed a three-steps process: rapid adsorption of metal ions, a transition phase, and a nearly flat plateau section. This process has also been confirmed by an intra-particle diffusion model (Figure 4).

Intraparticle diffusion was examined by plotting the amount of Cd^2+^ metal ions adsorbed against the square root of time. In this manner, the mechanism of the rate-limiting steps could be determined. As shown in Equation (1), the uptake for diffusion-controlled adsorption processes varies almost linearly with *t*^1/2^. The intra-particle diffusion rate constants were determined from the slope of the linear plot of *q_t_* *versus* *t*^1/2^, and the values of *C* were determined from the intercept. *C* provides an indication of the thickness or resistance of the boundary layer of adsorption. Figure 4 shows the second portion of intraparticle diffusion plot for Cd^2+^ onto JC at different initial metal ion concentrations. The graph also shows that the adsorption followed intraparticle diffusion after 2 h. It describes the gradual adsorption stage, which is the rate-limiting step.

The value of the rate constant for intra-particle diffusion, *K_id_* was calculated and found to increase from 0.0107 to 0.2418 mg·g^−1^·min^−0.5^ with increasing Cd^2+^ concentration from 2 to 50 ppm. The values of *C* increased from 0.2085 to 0.9551 mg/L with the square of correlation coefficient of 0.9249–0.9551, exhibiting an overall increase in the thickness and the effect of the boundary layer [5].

**Figure 5:**
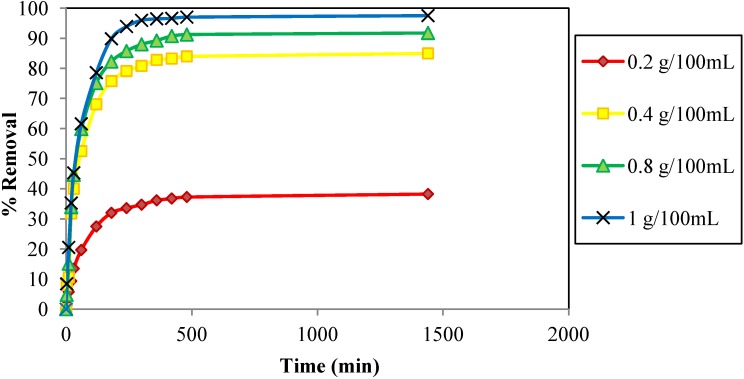
Effect of contact time on the adsorption of Cd^2+^ onto JC at various JC dosages. (adsorbent size: 0.5 mm, C_o_: 30 ppm, pH: 6, temperature: 25 °C, agitation speed: 160 rpm).

The rate of Cd^2+^ uptake was studied *versus* the adsorbent dose at 0.2–1 g/100 mL and at a constant Cd^2+^ concentration of 30 ppm, agitation speed of 160 rpm, pH 6.0, and contact time of 60 min. The uptake rate increased as the amount of adsorbent increased (Figure 5). The adsorption rate increased substantially when the dosages increased from 0.2 to 1 g/100 mL, this was due to the increase in the number of adsorption sites [5,6,34]. Maximum removal was observed at an adsorbent dose of 1 g/100 mL of JC. This confirms the notion that the adsorption rate is dependent on the amount of adsorbent. The gathered data for the Figure 5 were analyzed using Equation (2) and the values for percentage removal were plotted against the contact time, *t*, for the four different dosages.

**Figure 6:**
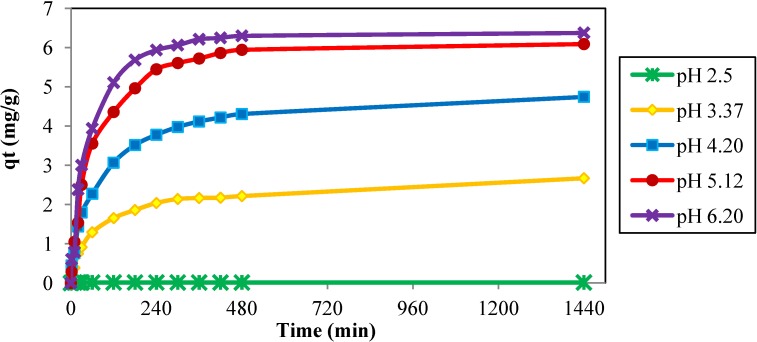
Effect of contact time on the adsorption of Cd^2+^ onto JC at different pH. (adsorbent size: 0.5 mm, C_o_: 30 ppm, dosage: 0.4 g/100 mL, temperature: 24 °C, and agitation speed: 160 rpm).

The pH of the medium has the greatest effect on controlling the uptake of adsorbates in aqueous solutions [34,35]. As shown in Figure 6, Cd^2+^ metal ion removal increased with each increment in the solution pH, with the maximum adsorption capacity occurring at pH 6. Adsorption did not occur at pH 2.5; this may have been due to the presence of hydrogen ions, which compete with the adsorption of Cd^2+^, and due to the chemical speciation of Cd^2+^ under the influence of the solution pH [5,6,28,30]. At very low pH, the repulsive force restricted the approach of metal cations to the surface ligands that were closely associated with the hydronium ions (H_3_O^+^). The nature of the adsorbent surface and the species distribution of the metal ions in the aqueous solution significantly affects metal ion adsorption. At very high pH, above 6.2, the precipitation occurs where the concentration of Cd^2+^ become very low due to insolubility of Cd(OH)_2_. Generally, the pH of Cd^2+^ from industrial waste are generated at pH lower than 7 [7].

In de-ionized water, cadmium species may be present in the forms of Cd^2+^, Cd(OH)^+^, Cd(OH)_2_, Cd(OH)_2_(S), *etc.*, and the concentration of the hydrolyzed cadmium species depends on the cadmium concentration and the solution pH [34].

Cd^2+^ + H_2_O ⇆ Cd(OH)^+^ + H^+^, p*K*_1_ = 7.9 (11)

Cd(OH)^+^ + H_2_O ⇆ Cd(OH)_2_ + H^+^, p*K*_1_ = 10.6 (12)

Cd(OH)_2_ + H_2_O ⇆ Cd(OH)_3_^−^ + H^+^, p*K*_1_ = 14.3 [34] (13)

Experiments were carried out at pH levels ranging from 2.5 to 6.2 to avoid the formation of hydroxyl complexes beyond pH 7 [5,6,34,35].

**Figure 7:**
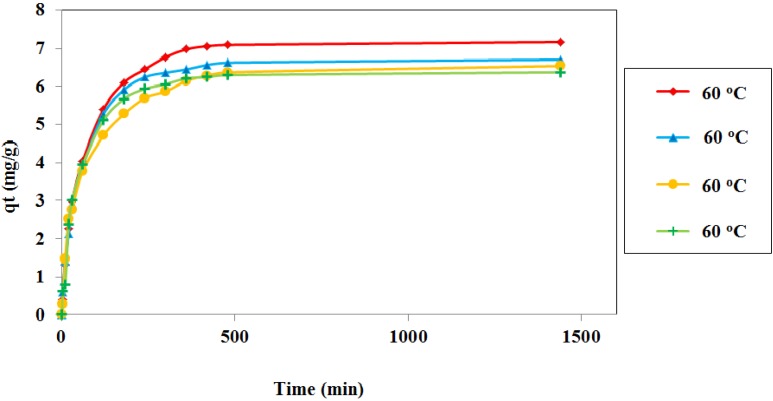
Effect of contact time on the adsorption of Cd^2+^ onto JC at different solution temperatures. (adsorbent size: 0.5 mm, C_o_: 30 ppm, dosage: 0.4 g/100 mL, pH: 6, and agitation speed: 160 rpm).

Temperature is an important parameter, as it exerts the greatest influence on the speed of adsorption processes. Indeed, an increase in temperature provides an increase in the amount of available energy and accelerates the adsorption process. Hence, elevated temperatures can reduce cost by decreasing the amount of time and adsorbent required for complete adsorption [4]. In this study, four solution temperatures (26, 35, 45 and 60 °C) and contact times up to 24 h were used. In all experiments, an adsorbent size of 0.5 mm, initial concentration of 30 ppm, dosage of 0.4 g/100 mL, pH 6, and agitation speed of 160 rpm were used. The gathered data were analyzed using Equation (3) and the values for *qt* were plotted against the contact time, *t*, for the four temperatures tested, as shown in Figure 7. The figures show that equilibrium was attained within 6–7 h for all systems. The results show that the adsorption of Cd^2+^ onto JC slightly increased from 6.37 to 7.17 mg/g with an increase in the temperature of the solution from 26 to 60 °C. The adsorbent derived from chlorella-based bio-mass also shows similar behavior. At a higher temperatures, the surface activity and kinetic energy were enhanced and increased the adsorption capacity [4,36].

### 3.3. Dynamic Adsorption Study

A dynamic adsorption study was carried out using the Lagergren pseudo-first-order model [(Equation (4)] and pseudo-second-order model [Equations (5)–(7)] to predict the mechanism involved in the adsorption process.

A plot of Log ( $q_{e^{-}}-q_{t}$ ) *versus t* produces a straight line for pseudo-first-order adsorption kinetics, which allows for the computation of the rate constant *K*_1_. Equation (6) was obtained by integrating and applying the following boundary conditions to Equation (5): *t* = 0 to *t* = *t* and *q* = 0 to *q* = *q_t_*. The term *dq*/*dt* represents the variation in adsorbate quantity with time. The values of *q_t_* and *q_e_* are as discussed above. The value of the constants *K_2_* (g·mg^−1^·min^−1^) and *q_e_* (mg/g) were calculated from a plot of *t/q_t_* *versus* *t*. As shown in Equation (7), the constant *K*_2_ was used to calculate the initial sorption rate, *h*, as *t*→ 0.

All kinetic parameters, including the correlation coefficients *R*^2^, were calculated and are presented in Table 1. The correlation coefficient value reveals that the adsorption of Cd^2+^ onto JC followed pseudo-second-order kinetics. The pseudo-second-order model for the removal of metal ions suggests that the adsorption basically followed a multi-step chemisorption process [5,6,33]. Moreover, Table 1 shows that the initial sorption rate *h* and the adsorption capacity increased with the initial metal ion concentration and solution pH, respectively. However, the initial sorption rate *h* becomes unpredictable with the increase in temperature and dosage; meanwhile, the adsorption capacity *q_e_* increases with temperature and decreases with dosage.

**Table 1:** Kinetic parameters for the adsorption of Cd^2+^ onto JC.

System parameters	Pseudo-first-order			Pseudo-second-order			
	*K*_1_	*q_e_*	*R*^2^	*K*_2_	*q_e_*	*h*	*R*^2^
**Initial concentration**							
0.5 ppm (mg/L)	0.0085	0.0394	0.8534	0.9453	0.0763	0.0055	0.9985
2 ppm (mg/L)	0.0090	0.3808	0.9736	0.0554	0.4589	0.0117	0.9921
10 ppm (mg/L)	0.0076	1.7195	0.9842	0.0110	2.2051	0.0536	0.9928
20 ppm (mg/L)	0.0081	3.5156	0.9861	0.0050	4.6860	0.1093	0.9934
30 ppm (mg/L)	0.0092	4.8362	0.9837	0.1477	6.7705	0.1913	0.9911
50 ppm (mg/L)	0.0067	7.6630	0.9892	0.1029	9.7182	0.2249	0.9913
**Initial Solution pH**							
pH 3.37	0.0037	2.0216	0.9026	0.0102	2.3883	0.0585	0.9936
pH 4.20	0.0048	3.6610	0.9650	0.0055	4.5558	0.1134	0.9904
pH 5.12	0.0076	4.9272	0.9870	0.0035	6.4309	0.1447	0.9925
pH 6.20	0.0092	4.8362	0.9837	0.0042	6.7705	0.1913	0.9911
**Temperature**							
26 °C	0.0053	5.4425	0.9202	0.0042	6.7705	0.1913	0.9911
35 °C	0.0090	5.2143	0.9859	0.0042	6.7522	0.1898	0.9938
45 °C	0.0071	5.1286	0.9880	0.0042	7.0522	0.2113	0.9949
60 °C	0.0092	4.8362	0.9837	0.0032	7.6687	0.1899	0.9939
**Dosage**							
0.2 g	0.0076	4.8040	0.8534	0.0031	6.1652	0.1177	0.9904
0.4 g	0.0092	4.8362	0.9736	0.0042	6.7705	0.1913	0.9911
0.8 g	0.0101	2.7340	0.9842	0.0080	3.6670	0.1071	0.9954
1.0 g	0.0113	2.2636	0.9861	0.0107	3.1114	0.1040	0.9958

### 3.4. Isotherm Study

The study of isotherms or adsorption equilibrium data is important in designing adsorption systems; the Langmuir and Freundlich isotherm models are commonly used in this respect [5,6]. Such isotherm studies help to determine the relationship between adsorbate concentration in the bulk, the uptake capabilities of the adsorbent under study, and the amount of species adsorbed at the adsorbent-solution interface [33,34]. In this study, adsorption equilibrium data were measured and fitted with the Langmuir and Freundlich isotherm equations for metal ion concentrations ranging from 0.5 to 50 ppm. The Langmuir model assumes that adsorption occurs uniformly at the active sites of the adsorbent or by monolayer adsorption and that no further adsorption can take place at filled sites. It describes adsorbent surfaces as being homogeneous and possessing an equally distributed adsorbate affinity; additionally, it holds that adsorption at one site will not affect adsorption at a nearby site [4]. Thus, the Langmuir isotherm equation was tested according to the initial metal ion concentrations [5,6,33]. Equation (8) illustrates the linearized form of the Langmuir isotherm. The Langmuir constants *q_m_* (maximum adsorption capacity, mg/g) and *K_L_* (parameter for Langmuir isotherm related to the affinity of the binding sites and energy of adsorption, L/mg) were predicted from the plot of 1/*q_e_* *versus* 1/*C_e_*. Equation (9) illustrates the Freundlich adsorption isotherm, which assumes that adsorption takes place on heterogeneous surfaces or through multilayer adsorption as opposed to monolayer adsorption.

The capacity and intensity of the adsorption were calculated from the intercept and slope of the plot of ln *q_e_* *versus* ln *C_e_* [5,6]. The fitted constants for the Freundlich and Langmuir models along with the associated regression coefficients are summarized in Table 2.

**Table 2:** Langmuir and Freundlich isotherm parameters for the adsorption of Cd^2+^ metal ions onto JC.

Freundlich			Langmuir			
*k_f_*	1/*n*	*R*^2^	*q_m_*	*k_L_*	*R_L_*	*R*^2^
0.7264	0.8332	0.9605	20.7900	0.0730	0.2152–0.8727	0.9976

**Figure 8:**
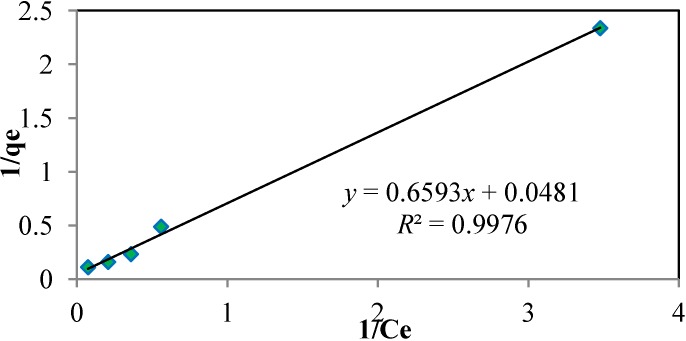
Linear plot of Langmuir isotherm for the adsorption of Cd^2+^ onto JC. (particle size: 0.5 mm, dosage: 0.4 g/100 mL, pH: 6, temperature: 24 °C, agitation speed: 160 rpm).

As can be seen from the regression coefficient data, the data conform to the Langmuir isotherm better; a linear plot is shown in Figure 8 with the Langmuir constants *q_m_* and *k_L_* assuming values of 20.79 mg/g and 0.73, respectively, for the adsorption of Cd^2+^ onto JC. This value for *q_m_* is higher compared to the values of some conventional and non-conventional adsorbents, such as physic seed hull (11.9 mg/g), castor hull (6.9 mg/g), granular activated carbon (1.39 mg/g), oak wood char (0.37 mg/g), pine bark char (0.34 mg/g), oak bark char (5.40 mg/g), carbon F-400 (8.00 mg/g), olive stone (0.9–9.72 mg/g), olive cake carbon (13.5 mg/g), perlite (0.42 mg/g), *Ceiba petandra* hull activated carbon (19.5 mg/g) and sawdust (41.21 mg/g) [6,7,33,37,38,39]. However, the value is lower compared to olive stone (128.2 mg/g) and sawdust (41.21 mg/g) [31,40]. The detailed of the comparison analysis has been tabulated in Table 3. The smaller particle size and low pH value could increase the adsorption rate, while, higher pH value would increase the possibility of precipitation of metal ions which could affect the results obtained. To obtain the optimum results, the concentration of 50 ppm, temperature of 60 °C, dosage of 1 g/100 mL, contact time of 6–7 h, and pH 6 should be used to generate higher adsorption capacity. However, in industrial applications, the consideration is influenced by the condition of the wastewater.

**Table 3:** Comparison of adsorption capacities (*q_m_*) of various adsorbents for removal of Cd^2+^.

No.	Adsorbents	*q_m_*	pH	Particle size (mm)	Temp. (°C)	Ref.
1	Physic seed hull	11.90	6	0.6	24	6
2	Castor hull	6.98	5.8	0.6	23	7
3	Granular activated carbon	1.39	5.8	1.18	23	7
4	Olive stone	128.2	4	0.25	24	31
5	Oak wood char	0.37	5	0.6–0.25	25	33
6	Pine bark char	0.34	5	0.6–0.25	25	33
7	Oak bark char	5.40	5	0.6–0.25	25	33
8	Carbon F-400	8.00	5	0.6–0.25	25	33
9	Perlite	0.42	6	2–1.7	22	37
10	Ceiba petandara hull a.c.	19.5	6	0.149	30	38
11	Sawdust	41.21	8	–	20	39
12	JC	20.79	6	0.5	24	Present work

The essential characteristics of the Langmuir isotherm can be expressed in terms of a dimensionless constant separation factor or equilibrium parameter, *R_L_*, which is illustrated in Equation (10). Dimensionless constant separation factor *R_L_* [40], *R_L_* is equal to the ratio of the unused adsorbent capacity to the maximum adsorbent capacity and indicates the shape of the isotherm as follows.

*R_L_* > 1, unfavorable,
*R_L_* = 1, linear,
0 < *R*_L_ <1, favorable,
*R_L_* < 0, irreversible [33,40].

**Figure 9:**
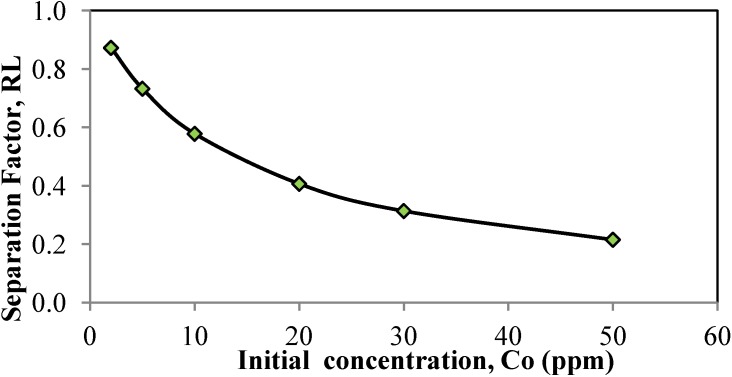
Separation factor, *R*_L_, of JC.

In this case, the *R*_L_ values were 0.2152–0.8727 (as shown in Figure 9), which fall between 0 and 1, indicating favorable adsorption. The *R*_L_ value was similar to that for the adsorption of Cd^2+^ metal ions onto *C. pentandra* hulls which indicates favorable adsorption [38].

### 3.5. Desorption Study

Desorption studies of Cd^2+^ ions were conducted to avoid the disposal of metal contaminated solid waste and to explore the possibility of reusage of JC as well as the recovery of the Cd^2+^ ions. HCl as chemical desorbent was chosen to eliminate the high cost of energy usage during thermal desorption. The total desorption for Cd^2+^ metal ions was 53%. The acid solution of 0.1 M HCl has a pH of 1, which allows the Cd^2+^ ions unattached from the surface ligands by the existence of hydrogen ions. The reduction of Cd^2+^ ions uptake by the reduction of pH also proved the phenomena (Figure 6). The 53% of Cd^2+^ ions retained in the JC was again performed, using the desorption process, several times, to measure the reusability of JC. There is only a 15% increment, of which 32% is still retained in the JC. The stronger acid solution and/or using of a complexing agent such as tartaric acid, ethylene diamine, and EDTA should be more effective on the removing and recovery of the Cd^2+^ ions from the used JC.

## 4. Conclusions

Jatropha seed hull carbon (JC) was successfully tested as an alternative adsorbent for the removal of Cd^2+^ metal ions from aqueous solutions. The adsorption capacity of JC was significantly influenced by the adsorbent surface characteristics and could be enhanced by increases in the contacts time, initial concentration, pH, temperature, and amount of adsorbent in the wastewater. The rate of sorption of metal ions was rapid during the initial 2–10 min, allowing for the removal of a major fraction of the adsorbate from the solution, and the adsorption reached equilibrium after 8 h. The results evidence the remarkable capacity of JC for Cd^2+^ metal ions adsorption, with a maximum monolayer adsorption capacity of 20.79 mg/g for Cd^2+^. The monolayer adsorption capacity (*q_m_*) of JC is comparable to the capacities of other reported agricultural-based adsorbents. The data reported herein are valuable for the design and fabrication of an economical treatment process using batched or stirred-tank flow reactors for the removal of metal ions from dilute industrial effluents.
